# Cox proportional hazard-model application: time to cervical cancer screening among women living with HIV in South Africa

**DOI:** 10.1186/s13027-023-00527-6

**Published:** 2024-03-02

**Authors:** Marcus Hollington

**Affiliations:** Demography and Population Studies, University of the Witwatersrand, School of Public Health & School of Social Sciences, Johannesburg, South Africa

**Keywords:** Cervical Cancer Screening, Human papillomavirus, Women Living with HIV, South Africa

## Abstract

**Background:**

There is an increased risk of cervical cancer among women living with HIV. While studies have long examined the association between cervical cancer among women with HIV, no study has examined the time taken for women with HIV to undergo cervical cancer screening as well as the hazard thereof in South Africa.

**Methods:**

The study used cross-sectional data from the 2016 South Africa Demographic and Health Survey. To allow for longitudinal analysis and to address the issue of right-censoring, the data were reformatted to a person-data file. The selection criteria were limited to women living with HIV (WLHIV) who had also responded to the question on cervical cancer screening. Descriptive statistics were employed to show the levels of HIV among women aged 15 and older in South Africa. Additionally, Kaplan‒Meier curves were employed to investigate the time to CCS by WLHIV in South Africa. Thereafter, an unadjusted Cox hazards regression model was employed to examine the hazard of undergoing CCS among WLHIV. Finally, it employed an adjusted model to examine the hazard of CCS among WLHIV while adjusting for other factors.

**Results:**

Nineteen percent (n = 1,159) of the women who participated in the study tested positive for HIV. Herein, it was found that the risk of CCS among WLHIV began at the age of approximately 19 years. Thereafter, the hazard of undergoing CCS among WLHIV began to decrease at 58 years. There was a significant association between CCS and WLHIV. Additionally, several covariates were found to be significantly associated with HIV. These were race, province, area of residence, marriage, educational attainment, employment, alcohol consumption, perceived health perception, and health insurance.

**Conclusion:**

The hazard of CCS was lower among WLHIV compared to WLHIV who did not undergo CCS in South Africa. This puts HIV-positive women at risk of increased morbidity and mortality from potential cervical cancer and HIV comorbidity due to CCS deficits within this group. This is because they are susceptible to HPV and subsequent cervical cancer due to a compromised immune system. HIV-positive women need to routinely undergo CCS every 12 months from baseline for 3 years. Thereafter, they should undergo CCS once every 3 years to reduce their risk of developing the disease.

## Background

The global prevalence of new HIV-attributable cervical cancer among women living with HIV (WLHIV) is 6% [1]. However, the regional differentials vary, disproportionately affecting WLHIV in Southern and Eastern Africa, constituting 64% and 27%, respectively [1, 2]. South Africa has one of the highest rates of human-immune virus (HIV) in the world. In 2017, an HIV household survey conducted in South Africa found that the country had a national HIV prevalence of 14%, translating to an estimated 8 million people living with HIV (PLHIV) [3]. Herein, gender disparities were observed in the distribution of HIV, with women constituting the greatest proportion of PLHIV at 51% and males constituting 46%. South Africa is considered the epicenter of the HIV pandemic in the world and is disproportionately affected by AIDS globally, with a fifth of all PLHIV residing in the country [4].

Moreover, the country is responsible for an estimated 20% of all new HIV infections in the world, with the incidence of HIV on the rise among adolescents with the odds of being HIV-positive being significantly higher among female adolescents compared to males (AOR = 2.24; P < 0.001; CI: 1.73–2.91) [4, 5]. Among women infected with HIV is the risk of contracting human papillomavirus (HPV), a precursor of cervical cancer [6]. Thus, there is a regular need to undergo cervical cancer screening (CCS) among HIV-positive women. In South Africa, several studies examining CCS among HIV-positive women have been conducted with varying findings [7–11]. The studies found that the factors associated with CCS among HIV-positive women include race, age, nationality, geography, marriage, educational attainment, cigarette smoking, and employment.

However, these studies have had a limited geographic scope, often focusing on hospitals, clinics, and provinces in an isolated manner, making it impossible to generalize studies nationally and provide country-wide policy recommendations to improve the uptake of CCS among WLHIV. To the author’s knowledge, no nationally representative study examining CCS among WLHIV has been conducted in South Africa. Additionally, to the author’s knowledge, no study has investigated the time taken to undergo CCS by WLHIV. This is particularly important given that WLHIV are highly susceptible to HPV and subsequent cervical cancer. The findings developed herein could better inform the cervical cancer prevention control policy (CCPCP) in South Africa, particularly its clause on CCS commencing from the age of 30 years, as it is perceived as the time to which the risk of cervical cancer is high [15]. The objectives of this study are to (a) highlight the level of HIV among women in South Africa, (b) investigate the time to CCS by WLHIV in South Africa, (c) ascertain the hazard of CCS among WLHIV, and (d) examine the hazard of CCS among WLHIV while adjusting for other factors.

## Methods

### Study design and setting

The study is a retrospective analysis of the 2016 South Africa Demography and Health Survey (SADHS). Permission to utilize the data was granted by the Demography and Health Survey program. South Africa was selected as the subject of analysis due to its disproportionate burden of HIV globally (20%) [4]. The SADHS is a nationally representative survey that is conducted in over 86 low- and middle-income countries every 5 years [12]. The survey is administered using a plethora of questionnaires. To obtain all variables of interest, including CCS and HIV, the study merged the adult women and HIV datasets using the one-to-one by observation type merge function in STATA 14.

### Study population and sample

The analysis was restricted to women aged 15 and older who responded to the question on HIV status. The total weighted sample constituted 6,126 women. However, after dropping participants who responded “don’t know” to the question on CCS and whose blood test results for HIV were inclusive, a final weighted sample of 6,067 was established. These were retrospectively assessed to determine the time (age in years) taken by WLHIV before they underwent CCS.

### Variable description

The outcome variable of this study is HIV status. It is dichotomous and categorized as (0) Negative and (1) Positive. It was derived from women aged 15 and older whose blood samples were tested for HIV during the study. Table 1 below shows the original variable name as presented in the HIV dataset of the SADHS, subsequent definition, and categorization as per the current study.

**Table 1:** Definition and categorization of dependent (outcome) variable

Variables	Original variable categories	Definition	Categorization
*Dependent (Outcome) Variable*			
HIV status	HIV negative	HIV blood test result	0. Negative
hiv03	HIV positive		
	Inconclusive		1. Positive

#### Covariates

The demographic, sociobehavioral, and health predictor variables are CCS, which constitutes itself as the mediating variable of this study, race, geography (namely, provinces and area of residence), marital status, educational attainment, employment, frequency of cigarette smoking, alcohol consumption, perception of own health, and health insurance.

#### Statistical analysis

The study employed cross-tabulations to illustrate the characteristics of the study participants. To allow for longitudinal analysis and to address the issue of right-censoring, the data were reformatted to a person-data file. In the context of this study, right-censoring refers to a case where women who underwent CCS would have not developed HIV by the end of the period of analysis or dropped out before the end of the analysis period. The advantage of the Cox proportional hazard model is that it does not include participants who did not experience the outcome of interest or dropped out from the study in the regression but includes them in the rest of the study. The results presented in this study account for censoring. After employing the Cox proportional hazard model, Kaplan‒Meier survival estimates were used to assess the time taken for women to develop HIV.

The formulas below were used to compute the survival estimates and generate the Kaplan‒Meier graphs in STATA 14:

$$S\left( {t_{i} } \right) = \coprod\limits_{{t_{i} < t}} {\left( {1 - \frac{{d_{i} }}{{n_{i} }}} \right)}$$

where $S({t}_{i}$) estimated probability of women developing HIV at time *t*, $ni$ number of women at risk of developing HIV at the beginning of time period ${t}_{i}$, ${d}_{i}$ number of HIV cases that occurred at time ${t}_{i}$.

The STATA commands below were executed to generate the graphs:

### stset AgeOfWomen, failure(HIVStatus)

#### *sts graph if AgeOfWomen *$\le$* 95, xlabel (15(7)(95)*

#### sts list

In this study, the period of the analysis is measured from age 15–95 years in which the subjects would or would not have developed HIV. Kaplan‒Meier graphs provide an illustration of the time taken to the development of HIV. Thereafter, the author employed a Cox hazard regression model to assess the influence that each covariate has on the development of HIV among women. Additionally, a second Cox hazard regression model was employed to assess the hazard of HIV among subjects while adjusting for other covariates. The study’s level of significance was set at P < 0.05. The unadjusted (Model 1) and adjusted (Model 2) results are reported as hazard ratios.

#### Model diagnostics

##### Test for multicollinearity

A test for multicollinearity was employed to examine the level of collinearity among study variables. This was done using a correlation matrix. A very high correlation coefficient above (0.80) between study variables was considered an indication of multicollinearity. The results of the correlation matrix presented in **Appendix A** indicated that there was no evidence of multicollinearity among the study variables.

## Results

### Sample characteristics

Table 2 below shows the weighted percentage distribution of the characteristics of the study participants. The full sample consisted of 6,067 women.

**Table 2:** Weighted distribution of study participants’ characteristics

Independent variables	Frequency	Percentage
Explanatory variable		
*CCS*		
Yes	2177	36
No	3890	64
Socio-demographic variables		
*Age*		
15–19	722	12
20–24	683	11
25–29	706	12
30–34	620	10
35–39	523	9
40–44	464	8
45–49	451	7
50 +	1898	31
*Race*		
Black/Africa	5128	85
White	256	4
Coloured	602	10
Indian, Asian and Other	81	1
*Province*		
Western Cape	474	8
Eastern Cape	791	13
Northern Cape	518	9
Free State	641	11
KwaZulu-Natal	965	16
North West	574	9
Gauteng	555	9
Mpumalanga	696	11
Limpopo	853	14
*Area of residence*		
Urban	3327	55
Rural	2740	45
*Marriage*		
Not married	3052	50
Married	3015	50
*Educational attainment*		
No education	569	9
Primary	1032	17
Secondary	3902	64
Higher	564	9
*Employment*		
Unemployed	4331	71
Employed	1736	29
Health/Risk factors		
*Cigarette smoking frequency*		
Does not smoke	5579	92
Every day	399	7
Some days	89	1
*Alcohol consumption*		
Yes	1562	26
No	4505	74
*Perception of own health*		
Poor	780	13
Average	2021	33
Good	2474	41
Excellent	792	13
*Health insurance*		
Yes	828	14
No	5239	86

The table shows the sample distribution of the study’s explanatory variable. Herein, 36% of women reported undergoing CCS at least once in their lifetime, while 64% reported never undergoing CCS in their lives. It also shows the sociodemographic characteristics of the sample. By age, women aged 50 and older constituted the greatest proportion of participants at 31%, followed by women aged 15–19 years at 12%, 25–29 years at 12%, 20–24 years at 11%, 30–34 years at 10%, 35–39 years at 9%, and 40–44 years at 8%, while women aged 45–49 years constituted the lowest proportion at 7%. In terms of provinces, women from KwaZulu-Natal constituted the greatest proportion of participants at 16%, followed by women from Limpopo at 14%, Eastern Cape at 13%, Mpumalanga at 11%, Free State at 11%, North West at 9%, Gauteng at 9%, and Northern Cape at 9%, while women from Western Cape constituted the least 8%.

By area of residence, women from urban areas constituted the greatest proportion at 55%, while women from rural areas constituted the least at 45%. In terms of marriage, women who reported being married and not being married constituted an equal proportion of 50% each. By educational attainment, women with secondary education constituted the greatest proportion of the sample at 64%, followed by women with primary education (17%), while women with no education and higher education both constituted 9%. By employment, unemployed women constituted the greatest proportion of the sample at 71%, while employed women constituted the smallest proportion at 29%.

The table concludes with the health/risk factor characteristics of the sample. In terms of frequency of cigarette smoking, women who reported that they did not smoke constituted the greatest proportion of the sample at 92%, followed by those who reported smoking every day at 7%, while women who reported smoking sometimes constituted the lowest proportion at 1%. By alcohol consumption, women who reported that they did not consume alcohol constituted the greatest proportion of the sample at 74%, while women who reported consuming alcohol constituted the lowest proportion at 26%.

**Fig. 1:**
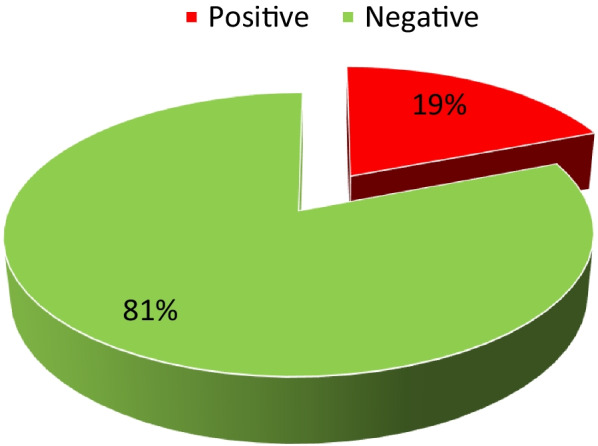
Levels of HIV among women aged 15 and older in South Africa

In terms of perception of their own health, women who reported being in good health constituted the greatest proportion of the sample at 41%, followed by women who reported their health as average (33%), while women who reported being in excellent and poor health both constituted the lowest proportion at 13%. Regarding health insurance, women who reported having no health insurance constituted the greatest proportion of participants at 86%, while women with health insurance constituted the smallest proportion at 14% (Fig. 1).

### Levels of HIV among women in South Africa

The study found that 19% of women aged between 15 years and older tested positive for HIV, while 81% of women in the sample tested negative for the disease.

### Time to CCS by WLHIV in South Africa

**Fig. 2:**
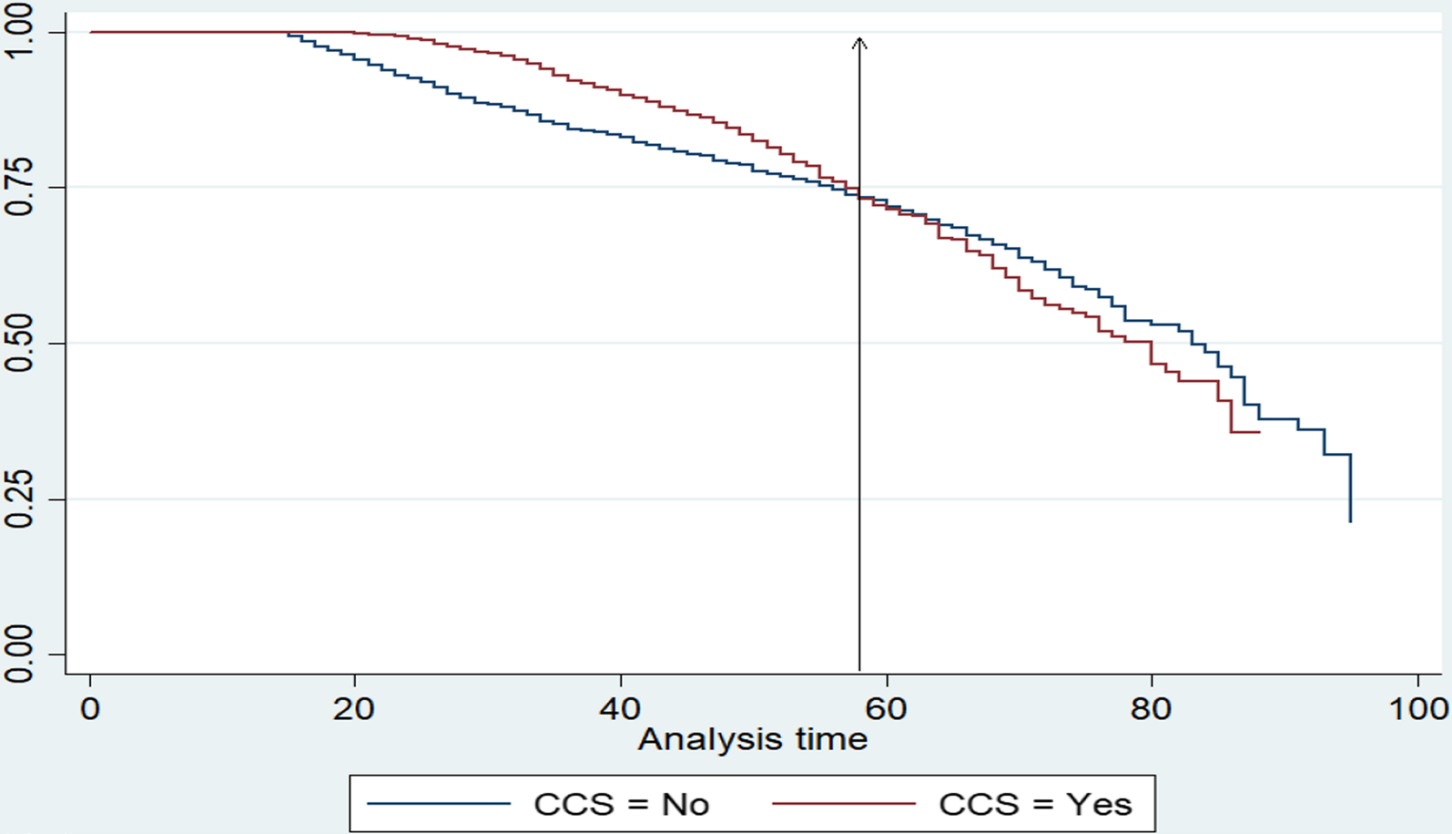
Time (age in years) to CCS among WLHIV in SA

Figure 2 below shows that the hazard of WLHIV undergoing CCS began at the age of approximately 19 years. Conversely, women who were diagnosed with HIV did not undergo CCS before the age of 19, as represented by the horizontal hazard line.

After the age of 19 years, the hazard line begins to change, showing that the event of interest (CCS) has occurred. The highest hazard of undergoing CCS among WLHIV was between the ages of 19–58. Approximately 74% of women within this age range had the probability of undergoing CCS. Thereafter, the hazard of undergoing CCS by WLHIV began to decrease at 58 years. In other words, the hazard of undergoing CCS started declining once WLHIV turned 58 years old. The hazard of undergoing CCS among WLHIV remained low from the age of 58 years. Conversely, the hazard of not undergoing CCS was higher among WLHIV from the age of 58 years and older compared to women who underwent CCS in the same age range. The hazard of not undergoing CCS among WLHIV dropped to approximately 73% at the age of 60 years.

### The hazard of CCS among WLHIV

Table 3 below shows the unadjusted and adjusted hazard ratios or risk of HIV by the characteristics of women aged 15 years and older. The effect of the study’s explanatory variable (CCS) was found to have a significant influence on HIV diagnosis. Herein, the study found that the hazard of CCS was 19% (Model 1) and 26% (Model 2) lower among WLHIV compared to women WLHIV who did not undergo CCS (UHR: 0.81; P < 0.05; CI: 0.72–0.91; AHR: 0.74; P < 0.05; CI: 0.65–0.85). Compared to Black/African women, the hazard of HIV diagnosis was decreased by 68% and 70% in Models 1 and 2, respectively, among women of white ethnicity (UHR: 0.42; P < 0.05; CI: 0.30–0.59; AHR: 0.30; P < 0.05; CI: 0.21–0.43). In terms of provinces, Model 2 showed that the hazard of HIV increased among women from the Free State (AHR: 1.47; P < 0.05; CI: 1.07–2.01) and KwaZulu-Natal (AHR: 1.45; P < 0.05; CI: 1.07–1.98) compared to women from the Western Cape.

**Table 3:** Effect of sample characteristics on HIV diagnosis among women

HIV	Model 1			Model 2		
	UHR	P Value	95% Confidence Interval	AHR	P Value	95% Confidence Interval
Explanatory variable						
CCS						
*No (R.C)*						
Yes	0.81	0.00***	0.72–0.91	0.74	0.00***	0.65–0.85
Sociodemographic variables						
Race						
*Black/African (R.C)*						
White	0.42	0.00***	0.30–0.59	0.3	0.00**	0.21–0.43
Coloured	1	0.97	0.82–1.20	1.09	0.5	0.84–1.41
Indian, Asian, and Other	0.7	0.18	0.41–1.18	0.67	0.17	0.39–1.18
Province						
*Western Cape (R.C)*						
Eastern Cape	1.09	0.52	0.84–1.43	1.34	0.06	0.99–1.82
Northern Cape	1.17	0.32	0.86–1.57	1.1	0.55	0.81–1.49
Free State	1.3	0.06	0.99–1.71	1.47	0.02***	1.07–2.01
KwaZulu-Natal	1.28	0.07	0.98–1.66	1.45	0.02***	1.07–1.98
North West	1.33	0.05	1.00–1.76	1.38	0.05	1.00–1.90
Gauteng	1.52	0.00***	1.15–2.02	1.47	0.02***	1.06–2.03
Mpumalanga	1.38	0.02***	1.05–1.82	1.73	0.00***	1.25–2.38
Limpopo	1.04	0.77	0.80–1.36	1.24	0.19	0.90–1.72
Area of Residence						
*Urban (R.C)*						
Rural	0.87	0.02***	0.78–0.98	0.95	0.49	0.82–1.10
Marriage						
*Not Married (R.C)*						
Married	0.43	0.00***	0.38–0.48	0.5	0.00***	0.44–0.57
Educational Attainment						
*No education (R.C)*						
Primary	1.77	0.00***	1.39–2.25	1.77	0.00***	1.39–2.26
Secondary	3.88	0.00***	3.13–4.81	4.18	0.00***	3.32–5.25
Higher	3.16	0.00***	2.41–4.15	4.44	0.00***	0.27–3.16
Employment						
*Unemployed (R.C)*						
Employed	1.38	0.00***	1.21–1.57	1.06	0.41	0.92–1.22
Health/Risk Factors						
*Frequency of smoking cigarettes*						
*Does not smoke (R.C)*						
Every day	0.89	0.33	0.70–1.13	0.97	0.83	0.75–1.26
Some days	1.28	0.28	0.82–1.99	1.2	0.43	0.76–1.89
*Alcohol consumption*						
*No (R.C)*						
Yes	1.2	0.01***	1.05–1.37	1.27	0.00***	1.11–1.46
Perception of own health						
*Poor (R.C)*						
Average	1.23	0.03***	1.02–148	1.19	0.08	0.98–1.44
Good	2.11	0.00***	1.76–2.53	1.9	0.00***	1.57–2.30
Excellent	2.84	0.00***	2.25–3.57	2.39	0.00***	1.87–3.04
Health insurance						
*No (R.C)*						
Yes	0.81	0.02***	0.68–0.96	0.75	0.01***	0.61–0.92

Conversely, compared to the Western Cape, the hazard of HIV increased among women from Gauteng (UHR: 1.52; P < 0.05; CI: 1.15–2.02; AHR: 1.47; P < 0.05; CI: 1.06–2.03) and Mpumalanga (UHR: 1.38; P < 0.05; CI: 1.05–1.82; AHR: 1.73; P < 0.05; CI: 1.25–2.38). By area of residence, Model 1 revealed that the hazard of HIV decreased by 13% among women from rural areas compared to women from urban areas. Compared to women who reported not being married, the hazard of HIV decreased by 57% and 50% in Models 1 and 2, respectively. By education, the hazard of HIV increased among women with primary (UHR: 1.77 & AHR: 1.77), secondary (UHR: 3.88 & AHR: 4.18), and higher education (UHR: 3.16 & AHR: 4.44) compared to women with no education.

Compared to unemployed women, the hazard of HIV increased among employed women (UHR: 1.38; P < 0.05: CI: 1.21–1.57). In terms of health/risk factors, women who reported consuming alcohol had higher risks of HIV than women who reported not consuming alcohol in both Models 1 and 2 of the study. Compared to women who perceived their own health as poor, the hazard of HIV increased with higher perceptions of personal health among WLHIV in both models. Finally, in terms of health insurance, the hazard of HIV decreased by 19% and 25% in Models 1 and 2, respectively, among women with health insurance compared to women who reported not having health insurance.

## Discussion

**Fig. 3:**
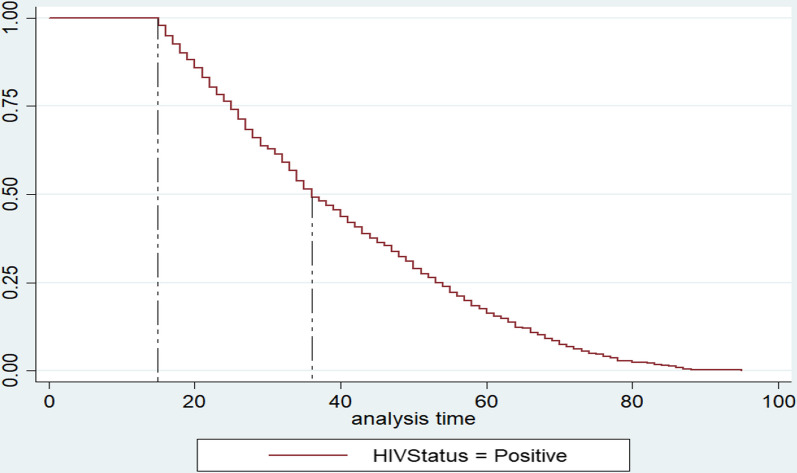
Time (age in years) to HIV infection among women in South Africa

The study found that young women in South Africa are also at risk of HIV. The age of risk begins at approximately 15 years old. This is consistent with the literature that suggests that adolescents in South Africa are highly susceptible to HIV (see Fig. 3) [5, 13, 14]. This risk of HIV was highest between 15 and 36 years old. Approximately 50% of women within this age range ran the risk of contracting HIV.

The important contribution that this study made was the time (age in years) taken by WLHIV to undergo CCS (see Fig. 3). This has never been done in South Africa and provides an opportunity to develop and improve the current cervical cancer prevention control policy (CCPCP) [15]. The CCPCP provides 3 government-sponsored cervical cancer screenings employed using a Pap smear test for women aged 30 years and older every decade [13, 15]. It has special age considerations for screening and treatment in cases where women have been diagnosed with HIV. However, what it does not have is a scientifically sound estimate of the CCS hazard among WLHIV that this study provides. The estimates provided in this study can be used to better improve the health outcomes of women diagnosed with HIV by providing insights pertaining to the time taken by WLHIV to undergo CCS in South Africa (see Fig. 3).

Moreover, as illustrated in Table 3, compared to women who did not undergo CCS, the hazard of women undergoing CCS decreased by 19% and 26%, respectively, among WLHIV, putting them at risk of contracting cervical cancer due to screening deficits. Thus, there is a need for awareness programs to educate women on the link between HIV and the development of cervical cancer to improve the uptake of CCS among WLHIV. The findings of this study broaden our understanding of health-seeking behaviour among WLHIV. The reason for early diagnosis of HIV among young women could be risky health behaviour such as alcohol consumption, which increased the hazard of women contracting HIV (UHR: 1.20; P < 0.05; CI: 1.05–1.37). This is consistent with the findings of other studies [7, 16].

In terms of geography, it was found that women from the Free State, KwaZulu-Natal, Gauteng, and Mpumalanga had increased risks of contracting HIV compared to women from the Western Cape (see Table 3). In Gauteng, the province’s increasing levels of urbanization could explain the increase in the hazard of HIV infections among women, especially considering the wealth disparities and levels of poverty in the province, which encourages risky sexual behaviour [17–19]. The increased hazards of HIV among women from Mpumalanga and KwaZulu-Natal were consistent with the findings of other studies highlighting the need for HIV intervention programs in these provinces, especially among adolescents [20]. Sociodemographic and economic factors such as race, marriage, employment, and education were associated with WLHIV and were consistent with the pertinent literature on the subject [7–11].

Interestingly, respondents’ perception of their own health was associated with HIV among women. Notably, the hazard of HIV increased with higher personal perceptions of health among women compared to those who reported perceiving their health as poor. This shows that women in South Africa should not assume excellent health without undergoing relevant tests to confirm, as this puts their health and that of others at great risk, further highlighting the need for HIV intervention programs among women to educate them on the importance of HIV screening to improve their health outcomes.

## Conclusion

The hazard of CCS is lower among WLHIV compared to women WLHIV who did not undergo CCS in South Africa. This puts WLHIV at risk of increased morbidity and mortality from potential cervical cancer and HIV comorbidity due to CCS deficits within this group, as they are susceptible to HPV and subsequent cervical cancer due to a compromised immune system. The study also found that the risk of HIV among adolescents who participated in this study began at the age of 15, highlighting the need for existing cervical cancer policies such as the CCPCP to include at-risk adolescents in government-funded CCS programs. Herein, it is recommended that the South African Department of Health extend its complementary screening services to include WLHIV younger than 30 years.

Additionally, it should shorten the CCS interval from 10 years to once every 12 months for the first 3 years at baseline, and if the results are normal, WLHIV should screen for cervical cancer once every 3 years to reduce their risk of developing HPV and subsequent cervical cancer [21, 22]. Early detection of CCS among WLHIV improves their health outcomes by reducing morbidity and mortality related to cervical cancer and HIV comorbidity. Ultimately improving their quality of life and life expectably by safeguarding WLHIV from premature death by preventable diseases such as cervical cancer.

## Data Availability

The datasets used and analyzed during the current study are available at the Demography and Health Survey program website https://dhsprogram.com/. The datasets can be accessed using the following login in credentials and project title “The relationship between domestic violence and cervical cancer screening among South African adults”: Email: hollington.marcus@gmail.com Password: #Chichi1%

## Appendix A

See Table

**Table 4:** Test for multicollinearity

Variables	(1)	(2)	(3)	(4)	(5)	(6)	(7)	(8)	(9)	(10)	(11)	(12)
(1) HIV Status	1.000											
(2) CCS	0.008	1.000										
	(0.535)											
(3) Race	− 0.005	0.175*	1.000									
	(0.695)	(0.000)										
(4) Province	0.008	− 0.141*	− 0.360*	1.000								
	(0.537)	(0.000)	(0.000)									
(5) Area Of Residence	− 0.007	− 0.173*	− 0.329*	0.325*	1.000							
	(0.580)	(0.000)	(0.000)	(0.000)								
(6) Married	− 0.008	0.226*	0.111*	− 0.013	− 0.024	1.000						
	(0.515)	(0.000)	(0.000)	(0.306)	(0.059)							
(7) EducationalAtt ~ t	0.006	0.125*	0.066*	− 0.035*	− 0.190*	− 0.171*	1.000					
	(0.640)	(0.000)	(0.000)	(0.006)	(0.000)	(0.000)						
(8) Employment	0.022	0.183*	0.067*	− 0.015	− 0.119*	0.066*	0.192*	1.000				
	(0.091)	(0.000)	(0.000)	(0.257)	(0.000)	(0.000)	(0.000)					
(9) CigaretteFrequ ~ e	0.003	0.056*	0.297*	− 0.171*	− 0.149*	0.058*	− 0.034*	0.020	1.000			
	(0.790)	(0.000)	(0.000)	(0.000)	(0.000)	(0.000)	(0.009)	(0.114)				
(10) EverConsumedL ~ r	− 0.003	0.065*	0.130*	− 0.115*	− 0.154*	− 0.009	0.103*	0.055*	0.190*	1.000		
	(0.800)	(0.000)	(0.000)	(0.000)	(0.000)	(0.472)	(0.000)	(0.000)	(0.000)			
(11) PerceptionOwn ~ h	− 0.006	− 0.062*	0.062*	0.038*	− 0.073*	− 0.141*	0.297*	0.080*	− 0.026*	0.026*	1.000	
	(0.649)	(0.000)	(0.000)	(0.003)	(0.000)	(0.000)	(0.000)	(0.000)	(0.039)	(0.044)		
(12) Health Insurance	− 0.016	0.208*	0.190*	− 0.077*	− 0.168*	0.105*	0.266*	0.200*	0.009	0.098*	0.095*	1.000
	(0.210)	(0.000)	(0.000)	(0.000)	(0.000)	(0.000)	(0.000)	(0.000)	(0.491)	(0.000)	(0.000)	

^***^* p* < *0.1, ** p* < *0.05, *** p* < *0.01*

*AHR* Adjusted Hazards Ratio, *CCS* Cervical Cancer Screening, *CCPCP* Cervical Cancer Prevention Control Policy, *HPV* Human papillomavirus, *PLHIV* People Living with HIV, *SADHS* South Africa Demography and Health Survey, *UHR* Unadjusted Hazards Ratios, *WLHIV* Women Living with HIV

4.

## Declarations

### Disclaimer

The article is an extract from a Master’s research report titled “The relationship between intimate partner violence and cervical cancer screening among women aged 15 years and older in South Africa" held by the Department of Demography and Population Studies, University of the Witwatersrand, Johannesburg, South Africa.

## References

[1] Stelzle D, Tanaka LF, Lee KK, Ibrahim Khalil A, Baussano I, Shah AS, Dalal S (2021). Estimates of the global burden of cervical cancer associated with HIV. Lancet Glob Health.

[2] World Health Organization. (2020). Who releases new estimates of the global burden of cervical cancer associated with HIV. Retrieved December 31, 2022, from https://www.who.int/news/item/16-11-2020-who-releases-new-estimates-of-the-global-burden-of-cervical-cancer-associated-with-hiv

[3] Zuma K, Simbayi L, Zungu N, Moyo S, Marinda E, Jooste S, Ramlagan S (2022). The HIV epidemic in South Africa: key findings from 2017 National Population-Based Survey. Int J Environ Res Public Health.

[4] Allinder, S., & Fleischman, J. (2020). The world's largest HIV epidemic in crisis: HIV in South Africa. Retrieved December 31, 2022, from https://www.csis.org/analysis/worlds-largest-hiv-epidemic-crisis-hiv-south-africa

[5] Mabaso M, Maseko G, Sewpaul R, Naidoo I, Jooste S, Takatshana S, Zungu N (2021). Trends and correlates of HIV prevalence among adolescents in South Africa: evidence from the 2008, 2012 and 2017 South African national HIV prevalence, incidence and behaviour surveys. AIDS Res Ther.

[6] National Cancer Institute. (2017). HIV infection and cancer risk. Retrieved December 31, 2022, from https://www.cancer.gov/about-cancer/causes-prevention/risk/infectious-agents/hiv-fact-sheet#:~:text=Because%20HIV-infected%20women%20have%20a%20higher%20risk%20of,with%20HIV%20infection%20up%20to%20age%2026%20years.

[7] Mokhele I, Evans D, Schnippel K, Swarts A, Smith JS, Firnhaber C (2016). Awareness, perceived risk and practices related to cervical cancer and pap smear screening: a cross-sectional study among HIV-positive women attending an Urban HIV clinic in Johannesburg, South Africa. South African Med J.

[8] Mabaso M, Maseko G, Sewpaul R, Naidoo I, Jooste S, Takatshana S, Zungu N (2021). Trends and correlates of HIV prevalence among adolescents in South Africa: evidence from the 2008, 2012 and 2017 South African national HIV prevalence, incidence and behaviour surveys. AIDS Res Ther.

[9] Lieber M, Afzal O, Shaia K, Mandelberger A, Du Preez C, Marie Beddoe A (2019). Cervical cancer screening in HIV-positive farmers in South Africa: mixed-method assessment. Ann Glob Health.

[10] Afzal O, Lieber M, Dottino P, Beddoe AM (2017). Cervical cancer screening in rural South Africa among HIV-infected migrant farm workers and Sex Workers. Gynecol Oncol Rep.

[11] Hollington M (2022). The association between intimate partner violence and cervical cancer screening among women of childbearing age: a South African Case Study. BMC Public Health.

[12] Demography and Health Survey. (n.d.). The DHS program. Retrieved December 31, 2022, from https://dhsprogram.com/Methodology/Survey-Types/DHS.cfm

[13] Hollington, M. (2022). *The relationship between intimate partner violence and cervical cancer screening among women aged 15 and older* (dissertation). Wiredspace, Johannesburg, South Africa.

[14] Steventon Roberts KJ, Sherr L, Haag K, Smith C, Jochim J, Toska E, Cluver L (2022). Adolescent parenthood and HIV-infection in South Africa—associations with Child Cognitive Development. PLOS Glob Public Health.

[15] NDoH. (2017). *Cervical Cancer Policy* (South Africa, National Department of Health, Department of Health).

[16] Godongwana M, De Wet-Billings N (2021). Time to hypertension development among people living with HIV in South Africa: a longitudinal analysis of the National Income Dynamics Survey (NIDS). Heliyon.

[17] Magadi MA (2016). Understanding the urban–rural disparity in HIV and poverty nexus: the case of Kenya. J Public Health.

[18] Van Schalkwyk C, Dorrington RE, Seatlhodi T, Velasquez C, Feizzadeh A, Johnson LF (2021). Modelling of HIV prevention and treatment progress in five South African metropolitan districts. Sci Rep.

[19] CoJ. (2019). *HIV/Aids in the City of Joburg: What the Data tells us in 2019* (South Africa, City of Johannesburg).

[20] Speizer IS, Xiong K, Mandal M, Makina-Zimalirana N, Hattori A, Durno D (2020). HIV-related knowledge, attitudes, and behaviors among grade 10 girls and boys in Mpumalanga and KwaZulu-Natal: Cross-sectional results. The Open AIDS J.

[21] Robbins HA, Strickler HD, Massad LS, Pierce CB, Darragh TM, Minkoff H, D'Souza G (2017). Cervical cancer screening intervals and management for women living with HIV. AIDS.

[22] Clinicalinfo. (2022). Human papillomavirus disease: NIH. Retrieved January 5, 2023, from https://clinicalinfo.hiv.gov/en/guidelines/hiv-clinical-guidelines-adult-and-adolescent-opportunistic-infections/human-0#:~:text=Women%20with%20HIV%20Aged%20%3C30%20Years&text=Pap%20test%20should%20be%20done,women%20younger%20than%2030%20years.

